# Transcoronary Ethanol Ablation For Ventricular Tachycardia

**Published:** 2011-11-15

**Authors:** Johnson Francis, Frederic Sacher

**Affiliations:** 1 KMCT Medical College, Calicut, Kerala, India; 2 Baby Memorial Hospital, Calicut, Kerala, India; 3 Univ Bordeaux II, Hospital Cardiologique du Haut-Leveque, Bordeaux-Pessac, France

**Keywords:** ventricular tachycardia, transcoronary ethanol ablation

Radiofrequency catheter ablation is a well established modality of treatment for ventricular tachycardia (VT). But it may not be successful in certain cases when the re-entrant circuit is situated deep within the myocardium and inaccessible to both endocardial and epicardial ablation. One of the earliest reports on transcoronary ethanol ablation of refractory ventricular tachycardia was by Inoue H et al in 1987 [1] in experimental animals followed by Brugada P et al in humans in1989 [2]. Brugada P et al identified the artery supplying the arrhythmogenic area and injected 1.5-6ml of sterile 96% ethanol, which cured the arrhythmia in two of the three patients. In the third one, arrhythmia recurred when a new collateral blood supply developed to the arrhythmogenic region, which was successfully treated by a repeat procedure. They had done a careful transcoronary mapping with saline prior to ablation in all cases. One of their patients developed temporary complete atrioventricular block after high septal ethanol ablation, requiring pacemaker.

Qi XQ et al [3] also reported successful transcoronary ethanol ablation in a patient with refractory ventricular tachycardia, who had no recurrence at two year follow up. They had demonstrated similar results in experimental animals earlier [4]. de Paola AA et al [5] documented ethanol ablation of ventricular tachycardia in chronic chagasic myocarditis. Programmed ventricular stimulation done after two weeks failed to induce ventricular tachycardia in their case. Nellens P et al [6] identified the culprit vessel by a combination of coronary angiography, left ventriculography, programmed electrical stimulation with endocardial mapping and pace mapping. Ethanol ablation was successful in all the ten of the twelve cases in which the artery could be identified. Six of them remained free of tachycardia and seven of them were alive, at a follow up period ranging from 2 to 44 months.

Verna E et al [7] described a case of fatal myocardial rupture and cardiac tamponade following successful alcohol ablation of refractory ventricular tachycardia and advised caution while proceeding with alcohol ablation.

Recent reports by  Sacher F et al [8] and Tokuda M et al [9] have once again highlighted the role of transcoronary ethanol ablation of refractory ventricular tachycardia after failed radiofrequency catheter ablation. Most of these were in the setting of ischemic cardiomyopathy with scar related re-entry. Reports of successful transcoronary ethanol ablation in ventricular tachycardias in hypertrophic cardiomyopathy [10] and ventricular tachycardia arising from the aortomitral continuity [11] are also available. Overall data would suggest that transcoronary ethanol ablation may be considered in refractory ventricular tachycardias which are not amenable to conventional radiofrequency catheter ablation due to deep seated foci of origin.

## References

[R1] Inoue H (1987). Intracoronary ethyl alcohol or phenol injection ablates aconitine-induced ventricular tachycardia in the dog. J Am Coll Cardiol.

[R2] Brugada P (1989). Transcoronary chemical ablation of ventricular tachycardia. Circulation.

[R3] Qi XQ (1992). Transcoronary chemical ablation of ventricular tachycardia. Chin Med J (Engl).

[R4] Qi XQ (1991). Transcoronary ethanol ablation of experimental ventricular tachycardia after epicardial ice mapping and localizing. Chin Med J (Engl).

[R5] de Paola AA (1992). Transcoronary chemical ablation of ventricular tachycardia in chronic chagasic myocarditis. J Am Coll Cardiol.

[R6] Nellens P (1992). Transcoronary chemical ablation of arrhythmias. Pacing Clin Electrophysiol.

[R7] Verna E (1992). Myocardial dissection following successful chemical ablation of ventricular tachycardia. Eur Heart J.

[R8] Sacher F (2008). Transcoronary ethanol ventricular tachycardia ablation in the modern electrophysiology era. Heart Rhythm.

[R9] Tokuda M (2011). Transcoronary Ethanol Ablation for Recurrent Ventricular Tachycardia After Failed Catheter Ablation: An Update. Circ Arrhythm Electrophysiol.

[R10] Inada K (2011). Substrate characterization and catheter ablation for monomorphic ventricular tachycardia in patients with apical hypertrophic cardiomyopathy. J Cardiovasc Electrophysiol.

[R11] Steven D (2009). Ventricular tachycardia arising from the aortomitral continuity in structural heart disease: characteristics and therapeutic considerations for an anatomically challenging area of origin. Circ Arrhythm Electrophysiol.

